# Block copolymer–based porous carbon fibers

**DOI:** 10.1126/sciadv.aau6852

**Published:** 2019-02-01

**Authors:** Zhengping Zhou, Tianyu Liu, Assad U. Khan, Guoliang Liu

**Affiliations:** 1 Department of Chemistry, Virginia Tech, Blacksburg, VA 24061, USA.; 2 Macromolecules Innovation Institute, Virginia Tech, Blacksburg, VA 24061, USA.; 3 Division of Nanoscience, Academy of Integrated Science, Virginia Tech, Blacksburg, VA 24061, USA.

## Abstract

Carbon fibers have high surface areas and rich functionalities for interacting with ions, molecules, and particles. However, the control over their porosity remains challenging. Conventional syntheses rely on blending polyacrylonitrile with sacrificial additives, which macrophase-separate and result in poorly controlled pores after pyrolysis. Here, we use block copolymer microphase separation, a fundamentally disparate approach to synthesizing porous carbon fibers (PCFs) with well-controlled mesopores (~10 nm) and micropores (~0.5 nm). Without infiltrating any carbon precursors or dopants, poly(acrylonitrile-*block*-methyl methacrylate) is directly converted to nitrogen and oxygen dual-doped PCFs. Owing to the interconnected network and the highly optimal bimodal pores, PCFs exhibit substantially reduced ion transport resistance and an ultrahigh capacitance of 66 μF cm^−2^ (6.6 times that of activated carbon). The approach of using block copolymer precursors revolutionizes the synthesis of PCFs. The advanced electrochemical properties signify that PCFs represent a new platform material for electrochemical energy storage.

## Teaser

The use of block copolymers as precursors revolutionizes the synthesis of porous carbon fibers with highly uniform pores.

## INTRODUCTION

**Fig. 1:**
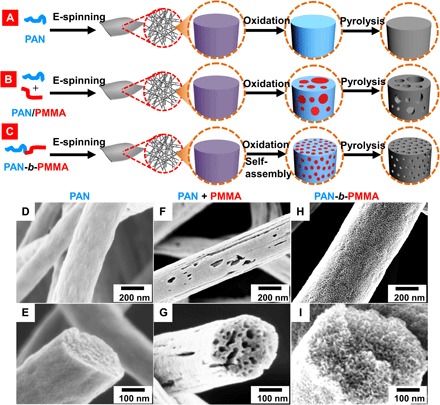
Fabrication of the carbon fibers. Conventional (**A** and **B**) and new (**C**) methods for synthesizing carbon fibers from various polymer precursors. (A) Pure PAN is electrospun into a fiber mat, oxidized at 280°C in air to crosslink PAN (blue), and then pyrolyzed at 800°C in N_2_ to generate carbon fibers (gray). An individual polymer fiber (purple) is magnified for illustration. (B) PAN is mixed with sacrificial PMMA (red) to form a polymer blend. After oxidation, the polymer blend macrophase-separates and forms nonuniform domains. After pyrolysis, PMMA is removed, resulting in nonuniform pores. (C) PAN-*b*-PMMA block copolymer microphase-separates into uniform PMMA nanodomains (red) in a matrix of PAN (blue) after oxidation and self-assembly. After pyrolysis, the PCFs contain well-controlled and uniformly distributed pores. SEM images of the carbon fibers from (**D** and **E**) PAN, (**F** and **G**) PAN/PMMA, and (**H** and **I**) PAN-*b*-PMMA.

Carbon fibers are superior materials for flexible and wearable electronics (*1*, *2*), as well as for aerospace and airspace applications (*3*), because of their outstanding mechanical strength, high flexibility, low density, excellent electrical conductivity, chemical stability, high temperature tolerance, and small thermal expansion coefficient (*3*–*5*). Carbon fibers mostly have been adopted as supporting scaffolds to alleviate the poor electrical conductivity of high-capacitance pseudocapacitive materials in supercapacitors (*6*–*9*). Besides serving as supports that contribute little to the supercapacitor capacitance, in the last decade, carbon fibers have been the focal point of growing efforts to turn them into active components (*1*, *10*, *11*). The direct use of carbon fibers as self-supporting electrodes removes the need for time-consuming processes to load other materials, such as pseudocapacitive materials, conductive additives, and binders (*12*, *13*), that introduce additional interfacial resistance and are detrimental to ultrafast charge/discharge. However, there are problems associated with this approach. Most carbon fibers are solid carbon filaments produced pyrolytically from pitch (*14*), polyacrylonitrile (PAN) (*15*) (Fig. 1, A, D, and E), and biorenewable polymers [e.g., cellulose (*16*), lignin (*17*), and others (*18*, *19*)]. Their smooth surfaces with limited effective surface areas (<10 m^2^ g^−1^) render them virtually incapable of storing a large amount of electrochemical energy (*20*, *21*). Therefore, strategies to increase the porosity of carbon fibers are highly desirable to realize their potential as active materials in electrochemical energy storage.

To increase their capacitance, carbon fibers must have (i) highly uniform pores (*22*) of certain sizes (preferably micropores of <1 nm and mesopores of ~10 nm) (*21*, *23*) and (ii) hierarchical porous structures to permit easy access by ions to the micropores (*24*). There are mainly two ways to create porous carbon fibers (PCFs). The first category is to posttreat carbon fibers via activation and chemical exfoliation (*25*, *26*). Activation with corrosive chemical agents (e.g., HNO_3_ and KOH) roughens the carbon fiber surfaces, but it usually needs highly reductive chemicals (e.g., hydrogen and hydrazine) and complicated postprocesses to restore the electrical conductivity. Chemical exfoliation is another facile way to activate carbon fibers, but with this method, it is difficult to control the porosity and pore size, as well as to preserve the fiber integrity.

The second strategy focuses on designing carbon fiber precursors. A variety of PCFs have been prepared by electrospinning PAN blended with sacrificial homopolymers (*27*, *28*), SiO_2_ nanoparticles (*29*, *30*), and carbon additives (*11*). Although these methods produce PCFs, their control over the pore size and uniformity is unsatisfactory because of the macrophase separation of the polymer blends (Fig. 1, B, F, and G, and fig. S1A) and the difficulty in distributing the additives uniformly in the PAN matrix. Furthermore, the removal of inorganic particles involves the use of highly toxic and corrosive chemicals, and often, it is challenging to fully remove the incorporated particles (*29*, *30*). Therefore, the development of an effective and efficient method for addressing the aforementioned obstacles, as well as the creation of uniform and hierarchical porous structures, is crucial to advance the electrochemical performance of carbon fibers (*22*).

The use of PAN-containing block copolymers as precursors is a promising way to create hierarchical porous structures in carbon fibers (*31*–*35*). In contrast to the polymer blends, block copolymers microphase-separate (*36*) to form highly uniform mesoporous structures (*34*, *37*, *38*) with pore sizes in the range of 2 to 50 nm (*39*–*42*). Furthermore, block copolymers can create interconnected mesopores and micropores. The hierarchical pores are important for high capacitive performance because mesopores serve as ion-buffering reservoirs and ion transport pathways which reduce the ion diffusion distances from the bulk electrolyte to the micropores, resulting in an enhanced rate capability (*6*, *21*, *22*).

Here, we present a method for synthesizing hierarchical PCFs with highly controlled structures by taking advantage of block copolymer self-assembly (Fig. 1, C, H, and I). Our method adopts a metal-free reversible addition-fragmentation chain transfer (RAFT) polymerization of poly(acrylonitrile-*block*-methyl methacrylate) (PAN-*b*-PMMA) followed by electrospinning, oxidation, and pyrolysis. The method is free from the use of etchants or activation chemicals. The as-prepared PCFs have hierarchically interconnected meso- and micropores with a high surface area of 503 m^2^ g^−1^ and a rich nitrogen content of 12.8%. The PCFs achieve a high surface area–normalized capacitance of 66 μF cm^−2^, substantially higher than any of the state-of-the-art PCFs derived from PAN, PAN blends, and other carbon fiber precursors (table S1).

## RESULTS

### Synthesis and structural characterization of hierarchical PCFs

**Fig. 2:**
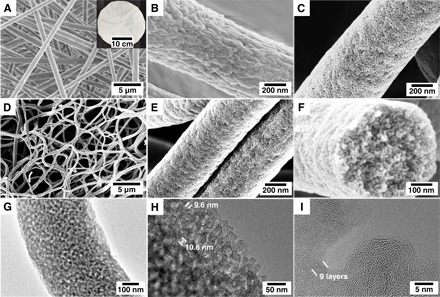
Microstructures of PCFs. SEM images of PAN-*b*-PMMA fibers (**A** and **B**) after electrospinning, (**C**) after oxidation and self-assembly at 280°C in air, and (**D** to **F**) after pyrolysis at 800°C in N_2_. Inset: A digital photograph of a piece of as-spun polymer fiber mat. (C) The bright and dark domains are PAN and PMMA, respectively. The good contrast between PAN and PMMA in the SEM image is due to the partial degradation of PMMA in air. (E and F) High-magnification SEM images of uniformly distributed mesopores in PAN-*b*-PMMA-CFs. (**G** and **H**) TEM images of mesopores in a PAN-*b*-PMMA-CF. (**I**) High-resolution TEM image of porous carbons at the edge of a PAN-*b*-PMMA-CF.

PAN-*b*-PMMA [111-*b*-62 kDa; polydispersity index (PDI), 1.14] with 64 volume % of PAN was synthesized and used as the carbon precursor. The as-electrospun polymer fibers were white and flexible (Fig. 2A). Because of the rapid solvent evaporation during electrospinning, the resulting polymer fibers had rough surfaces (Fig. 2B and fig. S1B). The average diameter of the polymer fibers was 911 ± 122 nm (fig. S1H). By crosslinking and cyclization of PAN at elevated temperatures in air (*43*), the oxidation process stabilized the fibrous structures (Fig. 2C and fig. S1C), and the fiber diameters did not change significantly afterward. The crosslinking of PAN prevented PAN-*b*-PMMA from forming long range–ordered nanostructures (*44*), and the resulting porous structures were interconnected and thus beneficial for ion transport in supercapacitors.

After oxidation, PAN-*b*-PMMA fibers exhibited microphase-separated poly(methyl methacrylate) (PMMA) domains in a PAN matrix (Fig. 2C and fig. S1C). PAN and PMMA showed excellent contrast and were easily distinguishable in the scanning electron microscopy (SEM) images, owing to the partial degradation of PMMA during the oxidation process as shown by the thermogravimetric analysis (TGA) (fig. S2). The thermally annealed polymer fibers were subjected to pyrolysis in an N_2_ atmosphere, resulting in PCFs termed PAN-*b*-PMMA-CFs (Fig. 2, D and E, and fig. S1D). Without the use of chemical exfoliation or activation, the pyrolysis of PAN-*b*-PMMA yielded a continuous porous carbon structure. The carbon yield was ~30.5% according to TGA (fig. S2). After pyrolysis, the average fiber diameter decreased to 519 ± 96 nm (fig. S1I) and the fibrous network remained intact. The carbon fiber diameter was substantially larger than the supposed diameter of 364 nm, assuming full pyrolysis and consolidation of polymers into non-PCFs (eq. S8), indicating that the resulting carbon fibers were highly porous and had a low density. The estimated porosity of carbon fibers was ~50.8%, in agreement with Brunauer-Emmett-Teller (BET) analysis (~50.6%; eqs. S9 and S10). The porous structure was confirmed by high-magnification SEM images (Figs. 1I and 2F and fig. S1D), in which mesopores were observed throughout the fibers. PAN-*b*-PMMA-CFs were flexible and remained intact when the fiber mat was bent to various angles (fig. S1, E to G).

Transmission electron microscopy (TEM) further confirmed the interconnected pores in the carbon fibers (Fig. 2, G to I). The mesopore size was determined to be ~10 nm, as highlighted in the TEM image (Fig. 2H). At the edge of the PCF, layered carbon structures were observed (Fig. 2I). The thickness of a single carbon layer (*d*_single_) was estimated by averaging over a number of carbon layers. For instance, the thickness of nine layers of carbon was ~3.46 nm (Fig. 2I), corresponding to an interplanar spacing of ~0.38 nm. The measured value agreed well with the value of ~0.37 nm calculated from x-ray diffraction (XRD) analysis. Both PAN-*b*-PMMA-CFs and PAN/PMMA-CFs (i.e., carbon fibers derived from PAN/PMMA) exhibited a full width at half maximum of the (100) peak of 0.11 in radian (fig. S3A and table S2, XRD section), which corresponded to a lateral size (*L*_a_) of ~2.8 nm according to the Debye-Scherrer equation (eq. S2). The lateral sizes of both PAN-*b*-PMMA-CFs and PAN/PMMA-CFs were twice that of PAN-CFs (carbon fibers derived from PAN). PAN-*b*-PMMA-CFs had a crystallite size (*L*_c_) of 0.98 nm, indicating about three to four π-stacked graphitic layers. In addition, Raman spectra of PAN-CFs, PAN/PMMA-CFs, and PAN-*b*-PMMA-CFs revealed the characteristic “G band” at ~1560 to 1600 cm^−1^ and “D band” at ~1310 to 1350 cm^−1^ (fig. S3B), corresponding to ordered graphitic structures and disordered domains, respectively. The calculated D band to G band intensity ratio (*I*_D_/*I*_G_) of all carbon fibers was 1.16 (table S2, Raman section), indicating that their graphitization degrees were identical.

**Fig. 3:**
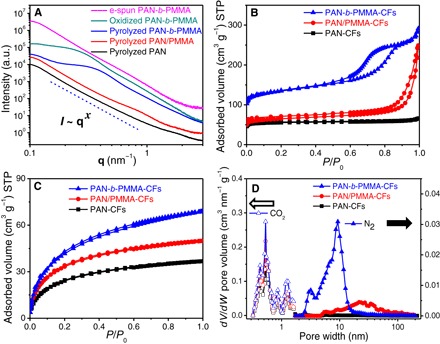
Porosity analyses of PCFs. (**A**) SAXS spectra of electrospun (e-spun), oxidized, and pyrolyzed PAN-*b*-PMMA fibers, in comparison with the carbon fibers pyrolyzed from PAN and PAN/PMMA blend. a.u., arbitrary units. (**B**) N_2_ at 77.4 K and (**C**) CO_2_ at 273.2 K adsorption-desorption isotherms of all carbon fibers. STP, standard temperature and pressure. (**D**) Calculated pore size distributions of all carbon fibers using NLDFT.

The microstructures of block copolymer and carbon fibers were further confirmed by small-angle x-ray scattering (SAXS; Fig. 3A). The SAXS spectrum of the as-electrospun PAN-*b*-PMMA fibers was devoid of any distinct features. After oxidation at 280°C, PAN-*b*-PMMA fibers exhibited a broad Bragg peak in the range of 0.172 to 0.218 nm^−1^, corresponding to center-to-center domain spacings of ~37 to 29 nm. The broad SAXS peak suggested that the microphase-separated structures of PAN-*b*-PMMA were likely disordered and lacked long-range order (*38*). After pyrolysis at 800°C, the position of the Bragg peak shifted to 0.224 to 0.308 nm^−1^ and the center-to-center spacings decreased to ~28 to 20 nm (table S2, SAXS section). The shift of the Bragg peak in SAXS spectra agreed with that in the fast Fourier transform (FFT) spectra (fig. S3C) from the SEM images (Fig. 2, C and E). FFT spectra showed center-to-center spacings of ~38 to 29 nm and ~29 to 20 nm for the oxidized and pyrolyzed PAN-*b*-PMMA fibers, respectively. In contrast, the pyrolyzed PAN and PAN/PMMA fibers did not exhibit any distinguishable features, indicating the absence of well-defined nanostructures. The scattering intensity (*I*) and wave vector (**q**) followed Porod’s law (*45*), *I* ~ **q**^*x*^. For PAN-*b*-PMMA fibers, the power-law index *x* ≈ −3.73, in contrast with a previous report (*35*) of *x* ≈ −4.00 for block copolymer powders. After pyrolysis, *x* increased to ~−3.43, suggesting that the surfaces of the carbon fibers were highly fractal and rough (table S2, SAXS section).

### Porosity analysis by gas adsorption-desorption isotherms

N_2_ and CO_2_ adsorption-desorption measurements of the PCFs (Fig. 3, B and C) revealed type IV and I isotherm characteristics, respectively. At relative N_2_ pressures (*P*/*P*_0_) of 0.6 to 0.9, the pronounced type H1 hysteresis of PAN-*b*-PMMA-CFs indicated the presence of mesopores according to the International Union of Pure and Applied Chemistry (IUPAC) classification (*46*). The pore size distribution was evaluated using a nonlocal density functional theory (NLDFT; Fig. 3D). PAN-*b*-PMMA-CFs had a BET surface area of 503 m^2^ g^−1^ (as calculated in the linear range of *P*/*P*_0_ = 0.01 to 0.1; Fig. 3B) and a total pore volume of 0.45 cm^3^ g^−1^ (table S2, BET section). Because of the block copolymer microphase separation, PAN-*b*-PMMA-CFs exhibited a considerable number of well-controlled mesopores with a narrow pore size distribution centered at ~9.3 nm (Fig. 3D). The SEM image analysis revealed an average mesopore size of ~10.1 nm (fig. S4, B to E), in good agreement with the average pore size obtained from the NLDFT model. Notably, similar to the pore size distribution obtained from the NLDFT model, the image analysis also showed a shoulder peak at ~3 to 4 nm (fig. S4E), validating the fact that the NLDFT model was suitable for pore size analysis of our PCFs. On the contrary, PAN/PMMA-CFs showed meso- and macropores with a much broader distribution (~2 to 200 nm), and the PAN-CFs had no detectable mesopores. Thus, PAN-*b*-PMMA provided substantially better control over the mesopore size than PAN and PAN/PMMA did. Furthermore, the uniform mesopores in PAN-*b*-PMMA-CFs offered numerous channels to interconnect with the micropores (peaked at 0.5 nm), leading to a 100% increase in the micropore volume (table S2, BET section). As a result, PAN-*b*-PMMA-CFs had a specific surface area of 503 m^2^ g^−1^, more than twice the surface areas of PAN-CFs (213 m^2^ g^−1^) and PAN/PMMA-CFs (245 m^2^ g^−1^). Taking together the SEM, SAXS, and BET results, we are confident that PAN-*b*-PMMA-CFs are equipped with a hierarchical porous structure, a feature that is indispensable to facilitate ion diffusion and to achieve high-rate capability in supercapacitors.

### Electrochemical performances and ion transport dynamics

**Fig. 4:**
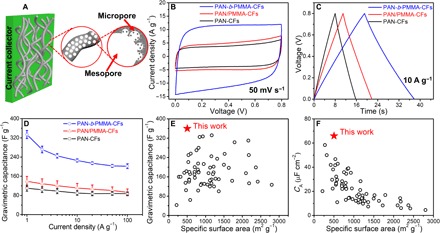
Electrochemical performance of the carbon fibers. (**A**) Schematic illustration of a binder-free and conductive additive–free electrode composed of PCFs. (**B**) CV curves at a scan rate of 50 mV s^−1^. (**C**) CP curves at a current density of 10 A g^−1^. (**D**) Gravimetric capacitance versus current density ranging from 1 to 100 A g^−1^. (**E**) Gravimetric capacitance and (**F**) BET surface area–normalized capacitance (*C*_A_) of PAN-*b*-PMMA-CFs at 1 A g^−1^, in comparison with those of other carbon fibers reported in the literature (black circles).

We postulate that the interconnected meso- and micropores in PAN-*b*-PMMA-CFs provide efficient pathways for rapid ion diffusion and enable outstanding capacitive performance (Fig. 4A). To test the capacitive performance, we assembled two-electrode symmetric supercapacitor cells. The electrodes were fabricated by sandwiching carbon fibers between two pieces of Ni foam without any conductive additives or polymer binders. PAN-*b*-PMMA-CFs exhibited nearly rectangular cyclic voltammograms with no discernible redox peaks, even at a high scan rate of 100 mV s^−1^, suggesting a near-ideal capacitive behavior (Fig. 4B and fig. S5A). In comparison with PAN/PMMA-CFs and PAN-CFs, PAN-*b*-PMMA-CFs had the largest area enclosed by cyclic voltammetry (CV) and thus the highest capacitance at a scan rate of 50 mV s^−1^ (Fig. 4B). Gravimetric capacitances were calculated based on chronopotentiometry (CP) (Fig. 4C and fig. S5C) and CV (fig. S5B). At 1 A g^−1^, the highest gravimetric capacitance of PAN-*b*-PMMA-CFs was 360 F g^−1^ and the average value over eight devices was 334 ± 17 F g^−1^. At a high current density of 10 A g^−1^, the gravimetric capacitance of PAN-*b*-PMMA-CFs reached 226 ± 6 F g^−1^ (Fig. 4D), twice those of PAN/PMMA-CFs (111 ± 19 F g^−1^) and PAN-CFs (90 ± 9 F g^−1^). At an extremely high current density of 100 A g^−1^, the capacitance was 202 ± 10 F g^−1^ and retained >60% of the value at 1 A g^−1^.

To further demonstrate the excellent electrochemical performances of the PCFs, we assembled and tested three-electrode cells in 6 M KOH aqueous solution. The electrochemical performance (fig. S5, E and F) corroborated that in a two-electrode testing configuration. When the current density was increased from 10 to 100 A g^−1^, the gravimetric capacitance of PAN-*b*-PMMA-CFs only dropped by 11%. The outstanding rate capability confirmed that the interconnected meso- and micropores in PAN-*b*-PMMA-CFs provided efficient pathways for rapid electrolyte infiltration and ion diffusion.

## DISCUSSION

PAN-*b*-PMMA-CFs exhibited an outstanding gravimetric capacitance of 360 F g^−1^ at a high current density of 1 A g^−1^ (Fig. 4E). This value is higher than those of the previously reported carbon fibers (table S1), as well as those of the previously reported nonfibrous porous carbons from PAN-containing block copolymers. For example, Zhong *et al*. (*35*) reported nanoporous nitrogen-enriched carbon derived from poly(acrylonitrile-*b*-butyl acrylate) with a gravimetric capacitance of ~166 F g^−1^ at 0.1 A g^−1^. Yan *et al*. (*34*) reported block copolymer–derived mesoporous carbon particles (in a nonfibrous format) as supercapacitor electrode materials with 254 F g^−1^ at 0.5 A g^−1^. Our PCFs displayed 33% better capacitive performance than KOH-activated carbon fibers, e.g., the N and O dual-doped, KOH-activated PCFs reported by Li *et al*. (*47*) with a capacitance of ~270 F g^−1^ at 1 A g^−1^. Our block copolymer–based PCFs were not subjected to any chemical activation or postsynthesis treatments. The enhanced capacitance of PAN-*b*-PMMA-CFs is mainly attributed to the fiber network and the well-defined hierarchical micro- and mesoporous structures. Without any binders, the pores provide continuous diffusion pathways for the ions, and the carbon matrix offers conduction pathways for the electrons (Fig. 4A).

**Fig. 5:**
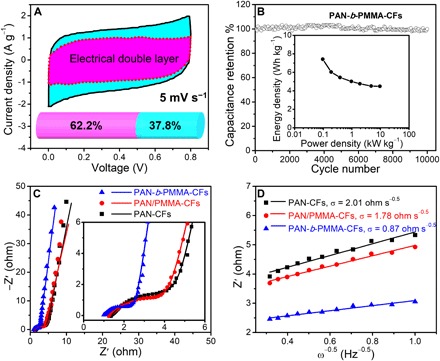
Electrochemical properties of the carbon fibers. (**A**) Dunn method analysis of capacitance contribution of PAN-*b*-PMMA-CFs. The shaded regions show the current contributions from the electrical double-layer capacitive (magenta) and pseudocapacitive (cyan) processes. Inset: A histogram shows the percentages of electrical double-layer capacitance (62.2%) and pseudocapacitance (37.8%). (**B**) Cycling stability of PAN-*b*-PMMA-CFs at a current density of 100 A g^−1^. Inset: Ragone plot of PCFs. The gravimetric energy density and the power density are calculated according to Eqs. 4 and 5, respectively. (**C**) Nyquist plots of carbon fibers in the frequency range of 100 kHz to 0.1 Hz with an across-current perturbation of 10 mV. Inset: Nyquist plots in the middle- and high-frequency range. The scattered points are experimental data, and the solid lines are the fitting curves. The impedances of the Nyquist plots are normalized to the working areas of the tested electrodes. (**D**) Linear fitting to the real part of impedance (*Z′*) versus the −1/2 power of the angular frequency (ω^−0.5^) plots in a frequency range of 1 to 10 Hz to extract the ion diffusion resistance (σ).

BET surface area–normalized capacitance (*C*_A_) of PAN-*b*-PMMA-CFs is as high as 66 ± 3 μF cm^−2^, higher than the previously reported PCFs and most porous carbons (Fig. 4F and table S1). We note that the *C*_A_ of PAN-*b*-PMMA-CFs is much higher than the typical value for electrical double-layer capacitance (5 to 20 μF cm^−2^) and 6.6 times that of activated carbon (10 μF cm^−2^) (*48*). The high capacitance stems from the pseudocapacitive reactions brought by the highly accessible heteroatoms (i.e., O and N). Two capacitance differentiation methods (Trasatti and Dunn’s methods; fig. S6) reveal that ~37% of the total capacitance is from pseudocapacitance (Fig. 5A). The PCFs display excellent long cycle life, as evaluated by the voltage holding tests (fig. S7) and the constant-current charge-discharge cycling test for 10,000 cycles (Fig. 5B). The PCF-based supercapacitors exhibit a power density of 9.6 kW kg^−1^ at an energy density of 4.5 Wh kg^−1^ (Fig. 5B, inset), notably higher than that of the commercially available supercapacitors (typically 1 to 2 kW kg^−1^ at a similar energy density of 5 Wh kg^−1^) (*49*).

Electrochemical impedance spectroscopy (EIS) reveals the ion and charge transport dynamics. In each Nyquist plot (Fig. 5C, fitted to an equivalent circuit model in fig. S5D), an incomplete semicircle in the high-frequency region is followed by a 45°-inclined Warburg diffusion line and a steep straight line in the low-frequency region. The equivalent circuit model (*50*) successfully describes the resistive features and the capacitive behavior of our PCF electrodes (fig. S5D). As shown in Fig. 5C, all carbon fibers exhibit nearly vertical low-frequency lines, indicating nearly ideal capacitive behavior (*51*). Among the three electrodes studied herein, PAN-*b*-PMMA-CFs exhibit the steepest linear line in the low-frequency range. This feature is correlated to the ion diffusion resistance, highlighting the merit of the hierarchical porous structures in PAN-*b*-PMMA-CFs and indicating that the ion diffusion in PAN-*b*-PMMA-CFs is the most efficient. To quantify the ion diffusion resistance, the Warburg coefficient σ (ohm s^−0.5^) can be extracted by fitting the real part of impedance (*Z′*) versus the −1/2 power of the angular frequency (ω^−0.5^) in the frequency range of 1 to 10 Hz (Fig. 5D). The slope of the fitted line equals the Warburg coefficient (σ), a parameter measuring the diffusion resistance when the ions diffuse through the electrodes (*52*). PAN-*b*-PMMA-CFs display a small σ value of 0.87 ohm s^−0.5^. In contrast, both PAN-CFs and PAN/PMMA-CFs exhibit high σ values of 2.01 and 1.78 ohm s^−0.5^, respectively (table S2, electrical properties section). The high-frequency *Z′* intercept and the incomplete semicircle are associated with equivalent series resistance (*R*_s_) and charge transfer resistance (*R*_ct_), respectively (Fig. 5C, inset) (*50*). PAN-*b*-PMMA-CFs exhibit an intercept and a semicircle smaller than those of PAN-CFs and PAN/PMMA-CFs, indicating lower *R*_s_ and *R*_ct_. These results prove that the uniformly distributed mesopores are advantageous for ion diffusion, and hence, PAN-*b*-PMMA-CFs display high capacitances and excellent rate capabilities.

To the best of our knowledge, this is the first report of highly uniform PCFs derived from block copolymers. Our results show that PAN-*b*-PMMA is a highly effective precursor to produce hierarchical meso- and micro-PCFs. In contrast to other carbon fiber precursors, PAN-*b*-PMMA requires no corrosive chemicals for postsynthesis activation, nor any additives to increase the surface area and to control the pore size. The narrow pore size distribution and high surface area of PAN-*b*-PMMA-CFs are ascribed to the microphase separation of the block copolymer. The mesopore size of PCFs can be fine-tuned by the polymer molecular weight. The mesopore size is expected to increase as the molecular weight of PMMA is increased. The change in mesopore size will further alter the porosity, surface area, and electrochemical performance of the PCFs.

As supercapacitor electrodes, PAN-*b*-PMMA-CFs exhibit performance superior to both PAN-CFs and PAN/PMMA-CFs. The high capacitive performance is due to the interconnected micro- and mesoporous carbon structures with high porosity, high surface area, and low resistance. The micropores of ~0.5 nm and the uniform mesopores of ~10 nm are favorable for high-capacitance ion storage (*21*–*23*) with two main advantages. First, the micropores on the mesopore walls provide highly ion-accessible surface areas to improve the electrochemical double-layer capacitance (Fig. 4A) (*24*). Second, the interconnected mesopores allow unperturbed ion transport and reduce the distances for ion diffusion from the bulk electrolyte to the micropores. In addition to the structural advantages, the hierarchical porous structure has a surface chemical composition with excellent electrolyte wettability, as revealed by x-ray photoelectron spectroscopy (XPS) (fig. S8 and table S2, XPS section). The gravimetric and area-normalized capacitances of PAN-*b*-PMMA-CFs are higher than any previously reported PCF electrodes. The ultrahigh values result from the synergistic effects of the interconnected hierarchical porous structures, the well-controlled pore sizes, and the electrochemically active nitrogen and oxygen functional groups that are easily accessible to ions (*24*). Because the PCFs are flexible, they are suitable for flexible electronics.

In summary, we demonstrate a disruptive method of using block copolymers for synthesizing PCFs with well-defined bimodal pores and outstanding electrochemical properties. PAN-*b*-PMMA produces PCFs via self-assembly and pyrolysis, eliminating the tedious postsynthesis steps that other template or chemical activation methods require. Moreover, PAN-*b*-PMMA offers remarkable control over the pore size uniformity, better than PAN/PMMA and other polymer blends. Importantly, the area-normalized capacitance of the PCFs reaches 66 μF cm^−2^, outperforming all previously reported carbon fibers, owing to their hierarchically interconnected meso- and micropores, rich nitrogen and oxygen contents, and self-supporting characteristics. Notably, our PCFs retain high capacitances at ultrahigh current densities, because the uniformly distributed mesopores facilitate ion diffusion across the fibers (*22*). The versatility of the method extends the frontier of PCF nanotechnology and enables the development of advanced applications beyond electrochemical energy storage, such as catalysis, separation, purification, and wearable sensors (*3*, *11*).

## MATERIALS AND METHODS

### Materials

Acrylonitrile (AN; ≥99%), methyl methacrylate (MMA; ≥99%), 2,2′-azobis(2-methylpropionitrile) (AIBN; ≥98%), cumyl dithiobenzoate (CDB; ≥99%), benzene (≥99.9%), aluminium oxide (activated, neutral, Brockmann Activity I), *N,N*-dimethylformamide (DMF; ≥99.7%), and dimethyl sulfoxide (DMSO; ≥99.9%) were purchased from Sigma-Aldrich. The monomers were passed through alumina columns to remove inhibitors before use. All other chemicals were used as received.

### Synthesis of PAN-*b*-PMMA block copolymer

According to our previous report, the PAN-*b*-PMMA block copolymer was synthesized by RAFT polymerization (*31*). First, a mixture of MMA (35.0 ml, 310 mmol), CDB (84.28 mg, 0.3094 mmol), and AIBN (25.42 mg, 0.1548 mmol) was dissolved in benzene (51.6 ml) in a 100-ml Schlenk flask. The mixture was subjected to three cycles of freeze-pump-thaw (FPT), followed by back-filling with N_2_. Then, the flask was placed in an oil bath at 60°C and stirred for 24 hours. The resulting PMMA macro–chain transfer agent (macro-CTA) was precipitated in methanol and dried under vacuum for 12 hours to completely evaporate the remaining solvent. The purified PMMA macro-CTA (*M*_n, SEC_ = ~62 kDa; PDI, 1.04) was used to synthesize PAN-*b*-PMMA block copolymers. PMMA macro-CTA (0.65 g, 11 μmol), AN (2.6 ml, 43 mmol), AIBN (0.44 mg, 2.7 μmol), and DMSO (7.22 ml) were mixed in a 40-ml Schlenk flask equipped with a magnetic stirring bar. The mixture was degassed by three FPT cycles and then heated in an oil bath at 65°C under N_2_ atmosphere for 24 hours. A PAN-*b*-PMMA block copolymer (*M*_n, SEC_ = ~173 kDa) with a PDI of 1.14 and a PAN volume fraction of 64% was obtained. The PAN-*b*-PMMA block copolymer was purified similarly to PMMA macro-CTA.

### Preparation of hierarchical PCFs

PAN-*b*-PMMA block copolymer was dissolved in DMF at a concentration of 14 weight %. The solution was stirred at 65°C for 2 hours and then electrospun to polymer fiber mats at a feeding rate of 1.5 ml/hour under a voltage of 18 kV (Acopian Technical Company, Easton, PA). The polymer fibers were collected on an in-house–built rotary Al disc. After electrospinning, the fiber mat was peeled off from the collector and dried in a vacuum oven at 60°C for 6 hours. Oxidation and pyrolysis of fibers were conducted in a tube furnace (Lindberg/Blue M, Asheville, NC). The fiber mat was first oxidized by heating from room temperature to 280°C at a rate of 1°C min^−1^ and kept at 280°C for 8 hours in air. After oxidation, the fiber mat was heated again from room temperature to 800°C at a rate of 10°C min^−1^, followed by carbonization at the same temperature for 1 hour in a N_2_ flow (200 standard cubic centimeters per minute). For comparison, pure PAN and a polymer blend of PAN and PMMA (denoted as PAN/PMMA; 64 volume % of PAN) were electrospun into polymer fiber mats and underwent the identical pyrolysis steps at 800°C to obtain carbon fiber mats.

### Electrochemical characterization

The electrochemical capacitive performance of the carbon fiber mats was evaluated in a symmetric two-electrode configuration. To assemble the testing electrodes, carbon fiber mats with a mass loading of at least 1.0 mg were sandwiched between two pieces of cleaned nickel foam without any conductive additives or binders. An aqueous KOH solution (6 M) was used as the electrolyte. The two electrodes were placed in the aqueous electrolyte. The distance between the two electrodes was ~2 cm to avoid any physical contact or electrical short circuit.

CV and EIS were performed on a PARSTAT 4000+ electrochemical workstation (Princeton Applied Research, AMETEK Inc.). CP experiments were carried out by a battery test system (model 580, Scribner Associates Inc.). The potential window chosen for both CV and CP was 0 to 0.8 V. EIS was conducted in a frequency range from 100 kHz to 0.1 Hz with a 10-mV perturbation. The electrochemical measurements of three-electrode cells were performed using a Gamry 600 (Gamry Instruments). The synthesized carbon fiber mat, a piece of bare nickel foam, and an Ag/AgCl (in saturated KCl) electrode were used as the working electrode, the counter electrode, and the reference electrode, respectively. The stability was evaluated by constant-current (100 A g^−1^) charge-discharge tests and voltage-holding tests. For the voltage-holding tests, the supercapacitors were charged at a current density of 4 A g^−1^, held at a maximum voltage of 0.8 V for 1 and 5 min, and then discharged at 4 A g^−1^. The charge-hold-discharge process was repeated for 20 cycles.

The gravimetric capacitance (*C* in F g^−1^) of supercapacitors was calculated using the discharge portion of the collected CP curves (*53*, *54*)

$$C=\frac{4I\Delta t}{m\Delta V}$$ (1)

where *I* is the discharge current (A), Δ*t* is the discharge time (s), Δ*V* is the potential window (V), and *m* is the sum of active material mass (g) of two electrodes.

Alternatively, *C* was evaluated from CV curves using the following equation

$$C=\frac{1}{2(V_{t}-V_{0})v}\int_{V_{0}}^{V_{t}}|I_{m}(V)|dV$$ (2)

where *I*_m_(*V*) is the current density (A g^−1^), *v* is the scan rate (mV s^−1^), and *V*_0_ and *V*_t_ are the lower and upper potential limits of the chosen potential window, respectively.

The average area-normalized capacitance (*C*_A_, μF cm^−2^) was calculated on the basis of the BET surface area according to the following formula

$$C_{A}=\frac{C}{A_{\text{BET}}}$$ (3)

where *A*_BET_ is the BET-specific surface area (m^2^ g^−1^).

The energy density (*E*, Wh kg^−1^) and power density (*P*, kW kg^−1^) of the supercapacitors were evaluated by using the following formulas

$$E_{\text{cell}}=\frac{1}{8}C\Delta V^{2}$$ (4)

$$P=\frac{E_{\text{cell}}}{\Delta_{t}}$$ (5)

## Supplementary Material

http://advances.sciencemag.org/cgi/content/full/5/2/eaau6852/DC1

## SUPPLEMENTARY MATERIALS

Supplementary material for this article is available at http://advances.sciencemag.org/cgi/content/full/5/2/eaau6852/DC1 Section S1. Characterization and instrumentation Section S2. Calculation of carbon fiber porosity using geometric analysis Section S3. Calculation of carbon fiber porosity using BET analysis Section S4. Calculation of the degree of mesopore interconnectivity Fig. S1. Additional SEM images, flexibility, and size distribution of PAN-*b*-PMMA-CFs. Fig. S2. Thermogravimetric analysis. Fig. S3. Wide-angle XRD spectra, Raman spectra, and FFT spectra. Fig. S4. Comparison of the pore size distributions from image analysis and NLDFT fitting. Fig. S5. Additional electrochemical performance of PAN-*b*-PMMA-CFs. Fig. S6. Capacitance contribution analyses. Fig. S7. Stability performance of PAN-*b*-PMMA-CFs. Fig. S8. XPS spectra and contact angles. Table S1. Summary of the electrochemical capacitive performance of PCF electrodes. Table S2. Summary of the physical and chemical characterization. Reference (*55*)
