# Supplementary material for: Block copolymer–based porous carbon fibers
Source: Sci Adv. 2019 Feb 1;5(2):eaau6852. doi: 10.1126/sciadv.aau6852 (PMC6358319; doi:10.1126/sciadv.aau6852)
Supplement: http://advances.sciencemag.org/cgi/content/full/5/2/eaau6852/DC1 [file supp_5_2_eaau6852__index.html]

Science Advances | Science Advances

## Supplementary Materials

**This PDF file includes:**

- Section S1. Characterization and instrumentation
- Section S2. Calculation of carbon fiber porosity using geometric analysis
- Section S3. Calculation of carbon fiber porosity using BET analysis
- Section S4. Calculation of the degree of mesopore interconnectivity
- Fig. S1. Additional SEM images, flexibility, and size distribution of PAN-*b*-PMMA-CFs.
- Fig. S2. Thermogravimetric analysis.
- Fig. S3. Wide-angle XRD spectra, Raman spectra, and FFT spectra.
- Fig. S4. Comparison of the pore size distributions from image analysis and NLDFT fitting.
- Fig. S5. Additional electrochemical performance of PAN-*b*-PMMA-CFs.
- Fig. S6. Capacitance contribution analyses.
- Fig. S7. Stability performance of PAN-*b*-PMMA-CFs.
- Fig. S8. XPS spectra and contact angles.
- Table S1. Summary of the electrochemical capacitive performance of PCF electrodes.
- Table S2. Summary of the physical and chemical characterization.
- Reference (*55*)

Download PDF

**Files in this Data Supplement:**

- Adobe PDF - aau6852\_SM.pdf
