# Supplementary material for: Block copolymer–based porous carbon fibers
Source: Sci Adv. 2019 Feb 1;5(2):eaau6852. doi: 10.1126/sciadv.aau6852 (PMC6358319; doi:10.1126/sciadv.aau6852)
Supplement: http://advances.sciencemag.org/cgi/content/full/5/2/eaau6852/DC1 [file aau6852_SM.pdf]

Science Advances
AAAS

advances.sciencemag.org/cgi/content/full/5/2/eaau6852/DC1

Supplementary Materials for

Block copolymer–based porous carbon fibers

Zhengping Zhou, Tianyu Liu, Assad U. Khan, Guoliang Liu*

*Corresponding author. Email: gliu1@vt.edu

Published 1 February 2019, *Sci. Adv.* **5**, eaau6852 (2019)
DOI: 10.1126/sciadv.aau6852

This PDF file includes:

Section S1. Characterization and instrumentation
Section S2. Calculation of carbon fiber porosity using geometric analysis
Section S3. Calculation of carbon fiber porosity using BET analysis
Section S4. Calculation of the degree of mesopore interconnectivity
Fig. S1. Additional SEM images, flexibility, and size distribution of PAN-*b*-PMMA-CFs.
Fig. S2. Thermogravimetric analysis.
Fig. S3. Wide-angle XRD spectra, Raman spectra, and FFT spectra.
Fig. S4. Comparison of the pore size distributions from image analysis and NLDFT fitting.
Fig. S5. Additional electrochemical performance of PAN-*b*-PMMA-CFs.
Fig. S6. Capacitance contribution analyses.
Fig. S7. Stability performance of PAN-*b*-PMMA-CFs.
Fig. S8. XPS spectra and contact angles.
Table S1. Summary of the electrochemical capacitive performance of PCF electrodes.
Table S2. Summary of the physical and chemical characterization.
Reference (*55*)

Section S1. Characterization and instrumentation

Characterization of polymers: The conversions of monomers were measured by ^{1}H NMR (400 MHz U4-DD2 Agilent spectrometer). The molecular weights of PAN, PMMA, and PAN-*b*-PMMA were measured by both ^{1}H NMR and size exclusion chromatography (SEC, EcoSEC HLC-8320, Tosoh Bioscience). The SEC was equipped with a multi-angle light scattering detector (miniDAWN TREOS, Wyatt), a UV detector, and a differential refractive index detector. DMF was used as the eluent and the flow rate was 0.5 mL/min. Polymer solutions in DMF (50 µL, 1 mg/mL) were injected into the SEC, and the traces were collected at 50 °C. The decomposition and pyrolysis profiles of PAN, PMMA, and PAN-*b*-PMMA were analyzed using a thermogravimetric analyzer (TA Instruments, TGA 5500) by heating the as-electrospun polymer fibers from the ambient temperature to 800 °C at a heating rate of 10 °C/min under an N_2 atmosphere.

X-ray diffraction (XRD): The crystallinity of the porous carbon fibers was examined using an X-ray diffractometer (MiniFlex 600, Rigaku, Cu K_α radiation, λ = 1.54 Å). The acceleration voltage and emission current were 40 kV and 15 mA, respectively. The XRD profiles were collected within a 2θ range from 10° to 80° at a scan speed of 0.3°/min and a step size of 0.05°. The interplanar spacing (d_{002}), the lateral size (L_a, also known as the in-plane crystal size), and the crystallite size (L_c) of the porous carbon fibers were calculated using the Bragg's law (Eq. 1) and Debye-Scherrer equations (Eqs. 2 and 3)

$$d_{002} = \lambda/2sin\theta \quad (1)$$

$$L_a = \frac{1.84\lambda}{\beta(10\text{l})cos\theta(10\text{l})} \quad (2)$$

$$L_c = \frac{0.89\lambda}{\beta(002)cos\theta(002)} \quad (3)$$

where θ and β are the diffraction angle and the full width at half maximum (FWHM) of diffraction peaks in radians, respectively. All the calculated values of these parameters for the carbon fibers are listed in table S2.

X-ray photoelectron spectroscopy (XPS): The chemical structures and elemental analyses of the porous carbon fibers were carried out on an X-ray photoelectron spectroscope (PHI VersaProbe III) under a pressure of 10^{-9} torr. The XPS spectra were acquired using monochromatic Al K_α X-

ray source (1486.6 eV) at 100 W over an area of 1400 × 100 μm^2 at an incident angle of 45°. The voltage step size was 1 eV for surveys and 0.1 eV for high-resolution scans. The dwell time at every step was 50 ms. All binding energies were referenced to adventitious C 1s at 284.8 eV. The chemical states of elements in the carbon fibers were assigned based on the PHI and NIST XPS databases. The atomic fraction of each element was calculated based on the area of each fitted peak.

Physisorption analysis: The surface area, absorbed volume and pore-size distribution (PSD) of carbon fibers were determined from N_2 (77.4 K) and CO_2 (273.2 K) adsorption-desorption isotherms using a Micromeritics-3Flex surface characterization analyzer. The surface area was calculated using a Brunauer-Emmett-Teller (BET) method in the linear range of P/P_0 = 0.01-0.1. The total pore volume was measured using a single point absorption at P/P_0 of ~0.99. The PSD was determined using non-local density functional theory (NLDFT). The micropore surface area and volume were calculated using the t-plot method (Harkins and Jura thickness equation) within the thickness range of 3.5 to 5.0 Å. Since the contribution from macropores was negligible for most carbon fibers (except the carbon fibers from PAN/PMMA blends), the mesopore area and volume were obtained by subtracting the micropore portions from the BET total surface area and total volume, respectively. The volume of macropores in porous carbon fibers derived from the PAN/PMMA blends was estimated using NLDFT.

Electron Microscopy: The as-electrospun polymer fibers, the oxidized fibers, and the pyrolyzed carbon fibers were imaged using a field-emission scanning electron microscope (SEM, LEO Zeiss 1550) at an acceleration voltage of 2 kV and a working distance of ~2-4 mm. The high-resolution transmission electron microscope (TEM, FEI Titan 300) operating at 300 kV was used to image the carbonaceous structures of carbon fibers.

Electrochemical impedance spectroscopy (EIS): The EIS was conducted on a PARSTAT 4000+ electrochemical workstation (Princeton Applied Research, AMETEK Inc.) in a frequency range from 100 kHz to 0.1 Hz with a 10-mV perturbation. The Nyquist plots were fitted with a selected equivalent circuit model (fig. S5D) using an EIS data analysis software (ZSimpWin). The equivalent series resistance (R_s) and charge transfer resistance (R_{ct}) were calculated based on fittings to the equivalent circuit model. The Warburg diffusion coefficients (σ, $\Omega\ s^{-0.5}$) were calculated by fitting of the real part of impedance (Z') versus the -1/2 power of the angular frequency ($\omega^{-0.5}$) in a frequency range of 1-10 Hz. The fitted resistances and coefficients are listed in table S2.

Raman analysis: Raman spectra were obtained on a Raman spectrometer (WITec alpha500 in combination with a Confocal Raman Microscope) in the range of 1000-1800 cm^{-1} at a laser excitation wavelength of 633 nm.

Small angle X-ray scattering (SAXS): SAXS was performed on a Bruker N8 Horizon (Cu K_α radiation, λ = 1.54 Å) at a generator voltage of 50 kV and a current of 1 mA. The Porod analyses were performed in the high-q range to extract the power index (x) of the Porod's Law, $I \sim q^x$. The extracted power indices are listed in table S2.

Contact angle measurement: The contact angles of porous carbon fiber mats were measured on a goniometer (KINO Industry Co. Ltd.) using a solution of 6 M KOH as the liquid of interest. The droplet size was set to be ~8-10 μL for consistency of the measurements.

Four-point probe measurement: The bulk resistivity of carbon fiber mats was measured using a four-point probe system (JANDEL RM3-AR). The bulk resistivity (ρ, Ω·cm) is described as follows

$$\rho = 2\pi S \frac{V}{I} \tag{4}$$

where S is the probe spacing (0.1 cm), V is the voltage (V) and I is the current (A).

Section S2. Calculation of carbon fiber porosity using geometric analysis

If the polymer fibers are fully consolidated to non-porous carbon fibers (NPCF) after pyrolysis, the diameter of the resulting non-porous carbon fibers can be estimated based on the densities of the polymers and carbon, the volumes of the polymer and carbon fibers, and the carbon yield (30.5%, as measured with thermogravimetric analysis (TGA)). In principle, the total mass of carbon should be balanced as follows

$$\rho_{polymer} V_{polymer} \times 30.5\% = \rho_{carbon} V_{carbon} \tag{5}$$

where the volume of the polymer fibers can be calculated assuming a fiber length of $L_{polymer}$

$$V_{polymer} = \pi \times (\frac{d_{polymer}}{2})^2 \times L_{polymer} \tag{6}$$

and the volume of the non-porous carbon fibers can be similarly calculated assuming a fiber length of L_{NPCF}

$$V_{NPCF} = \pi \times (\frac{d_{NPCF}}{2})^2 \times L_{NPCF} \tag{7}$$

where $\rho_{polymer}$, $d_{polymer}$, $L_{polymer}$, and $V_{polymer}$ are the density, diameter, length, and volume of the block copolymer fibers, respectively; ρ_{NPCF}, d_{NPCF}, L_{NPCF}, and V_{NPCF} are the density, diameter, length, and volume of the non-porous carbon fibers, respectively.

According to the SEM images, the average diameter of PAN-*b*-PMMA fibers is 911 ± 122 nm. The densities of the polymer and carbon are 1.18 and 2.25 g/cm^3, respectively. Assuming the length of fibers remains the same before and after pyrolysis ($L_{polymer} \approx L_{NPCF}$), the diameter of non-porous carbon fibers can be estimated as follows

$$d_{NPCF} = \sqrt{\frac{\rho_{polymer} \times (d_{polymer})^2 \times 30.5\%}{\rho_{NPCF}}} = 364 \text{ nm} \tag{8}$$

According to the SEM images, the measured diameter of the porous carbon fibers (d_{PCF}) is 519 ± 96 nm. Thus, the porosity ($\emptyset_{pore}$) of the porous carbon fibers (PCF) can be estimated by the fraction of pore volume in the measured carbon fibers, as follows

$$\emptyset_{pore} = 1 - \frac{V_{NPCF}}{V_{PCF}} \times 100\% = 1 - \frac{{d_{NPCF}}^2}{{d_{PCF}}^2} \times 100\% = 50.8\% \tag{9}$$

where V_{PCF} is the volume of non-porous carbon fibers.

Section S3. Calculation of carbon fiber porosity using BET analysis

In addition to the geometric analysis, Brunauer-Emmett-Teller (BET) measurements can also be used to estimate the porosity of PAN-*b*-PMMA-CFs. Using values obtained from BET analysis, the porosity ($\emptyset_{BET}$) of PAN-*b*-PMMA-CFs can be calculated as

$$\emptyset_{BET} = \frac{V_{BET}}{V_{BET}+V_{NPCF}} \times 100\% = \frac{0.45}{0.45+1/2.25} \times 100\% = 50.6\% \tag{10}$$

where V_{BET} is the total pore volume of PAN-*b*-PMMA-CFs measured by BET (as shown in table S2), V_{NPCF} is the total carbon volume based on carbon density (2.25 g/cm^3). The porosity of PAN-*b*-PMMA-CFs after pyrolysis at 800 °C was calculated to be ~50.6%, in excellent agreement with that determined using the geometric analysis (50.8%).

Section S4. Calculation of the degree of mesopore interconnectivity

Since the pyrolysis of PAN contributes little to the mesopore volume, as evidenced by the pore-size distributions (PSDs) of PAN-CFs (Fig. 3D), we assume that the mesopores mostly arise by the removal of PMMA. In addition, assuming that all mesopores generated by PMMA are interconnected, we can calculate the theoretical total mesopore volume using the mass fraction of PMMA (ψ_{PMMA}). ψ_{PMMA} can be determined using the following equation

$$\psi_{\mathrm{PMMA}} = \frac{M_{n,\mathrm{PMMA}}}{M_{n,\mathrm{PMMA}}+M_{n,\mathrm{PAN}}} \times 100\% = \frac{62.0\ \mathrm{kDa}}{111\ \mathrm{kDa}+62.0\ \mathrm{kDa}} \times 100\% = 35.8\% \qquad (11)$$

where $M_{n,\mathrm{PMMA}}$ and $M_{n,\mathrm{PAN}}$ are the number-averaged molecular weights of PMMA and PAN, respectively, as determined by SEC.

In 1 g of PAN-*b*-PMMA, the mass of PMMA is

$$m_{\mathrm{PMMA}} = m_{\mathrm{BCP}} \times \psi_{\mathrm{PMMA}} = 1\ \mathrm{g} \times 35.8\% = 0.358\ \mathrm{g} \qquad (12)$$

where m_{BCP} is the total mass of PAN-*b*-PMMA. Note that the mass of the block copolymer is arbitrary and its value does not alter the final conclusion. We chose 1 g for simplicity.

Converting m_{PMMA} to volume, we have

$$V_{\mathrm{PMMA}} = \frac{m_{\mathrm{PMMA}}}{\rho_{\mathrm{PMMA}}} = \frac{0.358\ \mathrm{g}}{1.18\ \mathrm{g\ cm^{-3}}} = 0.303\ \mathrm{cm^3} \qquad (13)$$

where the density of PMMA is $\rho_{\mathrm{PMMA}} = 1.18\ \mathrm{g\ cm^{-3}}$. The char yield of PAN-*b*-PMMA is 30.5% according to TGA (fig. S2A). Thus, the carbon from 1 g of PAN-*b*-PMMA is

$$m_C = 30.5\% \times 1\ \mathrm{g} = 0.305\ \mathrm{g} \qquad (14)$$

Because the block copolymer fibers shrink significantly after pyrolysis, the mesopores shrink accordingly. The percentage of the volumetric shrinkage ($V_{\text{shrink}}\%$) can be estimated by the difference in fiber diameters, assuming that the length of the fibers remains unchanged during the pyrolysis

$$V_{shrink}\% = \frac{V_C}{V_{BCP}} \times 100\% = \frac{d_c^2}{d_{BCP}^2} \times 100\% = \frac{(519\ \text{nm})^2}{(911\ \text{nm})^2} \times 100\% = 32.5\% \tag{15}$$

where V_C, V_{BCP}, d_C and d_{BCP} are carbon fiber volume, block copolymer fiber volume, carbon fiber diameter, and block copolymer fiber diameter, respectively. Therefore, the theoretical mesopore volume is

$$V_{\text{mesopore,theo}} = V_{shrink}\% \times \frac{V_{\text{PMMA}}}{m_C} = 32.5\% \times \frac{0.303\ \text{cm}^3}{0.305\ \text{g}} = 0.323\ (\text{cm}^3\ \text{g}^{-1}) \tag{16}$$

The experimentally measured mesopore volume ($V_{\text{mesopore,exp}}$) is 0.310 $\text{cm}^3\ \text{g}^{-1}$ (table S2, BET section). Comparing the theoretical value with the measured value, the degree of mesopore interconnectivity (η) is calculated to be 96.0%, indicating that the majority of the mesopores are interconnected

$$\eta = \frac{V_{\text{mesopore,exp}}}{V_{\text{mesopore,theo}}} = \frac{0.310\ \text{cm}^3\ \text{g}^{-1}}{0.323\ \text{cm}^3\ \text{g}^{-1}} = 96.0\% \tag{17}$$

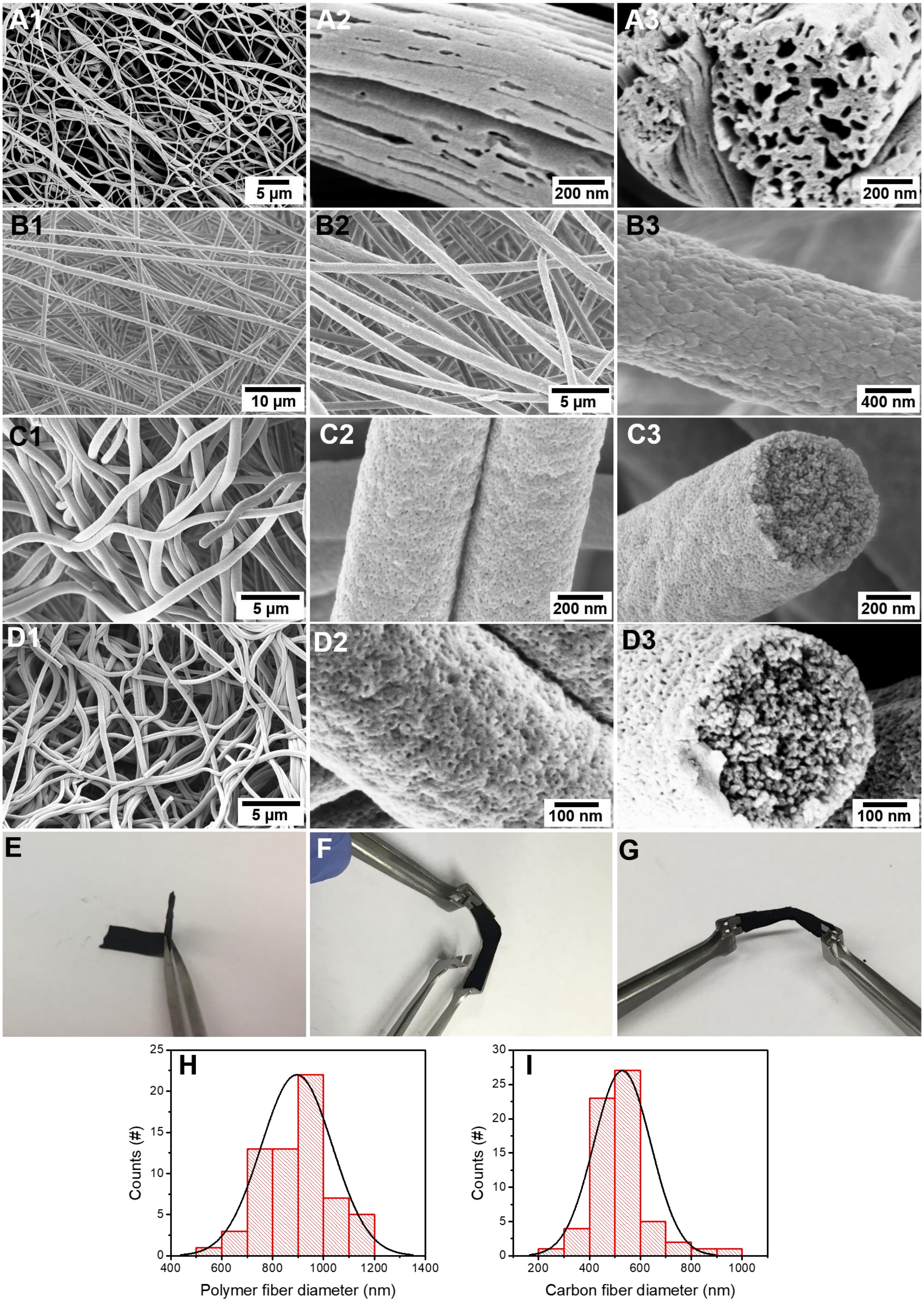
A1
5 µm
A2
200 nm
A3
200 nm
B1
10 µm
B2
5 µm
B3
400 nm
C1
5 µm
C2
200 nm
C3
200 nm
D1
5 µm
D2
100 nm
D3
100 nm
E
F
G
H
Counts (#)
Polymer fiber diameter (nm)
0
5
10
15
20
25
400
600
800
1000
1200
1400
I
Counts (#)
Carbon fiber diameter (nm)
0
5
10
15
20
25
30
200
400
600
800
1000

Fig. S1. Additional SEM images, flexibility, and size distribution of PAN-*b*-PMMA-CFs. (A) Additional SEM images of porous carbon fibers from a binary polymer blend of PAN and PMMA. The ratio of PAN:PMMA was 64:36 by volume, matching that of the PAN-*b*-PMMA block copolymer. Non-uniform porous structures were observed after pyrolysis at 800 °C. (B-D) Additional SEM images of (B) as-electrospun PAN-*b*-PMMA block copolymer fibers at various magnifications, (C) PAN-*b*-PMMA fibers after oxidation at 280 °C in air, and (D) PAN-*b*-PMMA-CFs after pyrolysis at 800 °C. (E-G) Photographs of a piece of PAN-*b*-PMMA-CFs bent at various angles. Statistic histograms of the diameters of (H) the as-electrospun PAN-*b*-PMMA block copolymer fibers and (I) the corresponding porous carbon fibers after pyrolysis.

The PAN-*b*-PMMA fibers were prepared by single-spinneret electrospinning. For most block copolymers, coaxial electrospinning is required to provide additional protective sheath layers so that the fibers can maintain a fibrous structure and survive the subsequent thermal annealing. For the PAN-*b*-PMMA block copolymer, because PAN can self-stabilize and crosslink to form ladder structures at elevated temperatures, no sheath material was required during the electrospinning to maintain the fibrous structure and survive the subsequent oxidation and pyrolysis steps.
During oxidation, PAN self-stabilized and crosslinked into a ladder molecular structure, which was critical to maintain the integrity of the fibrous structures after pyrolysis. In addition, due to the microphase separation of PAN and PMMA, the PAN-*b*-PMMA block copolymer self-assembled into well-defined nanostructures.

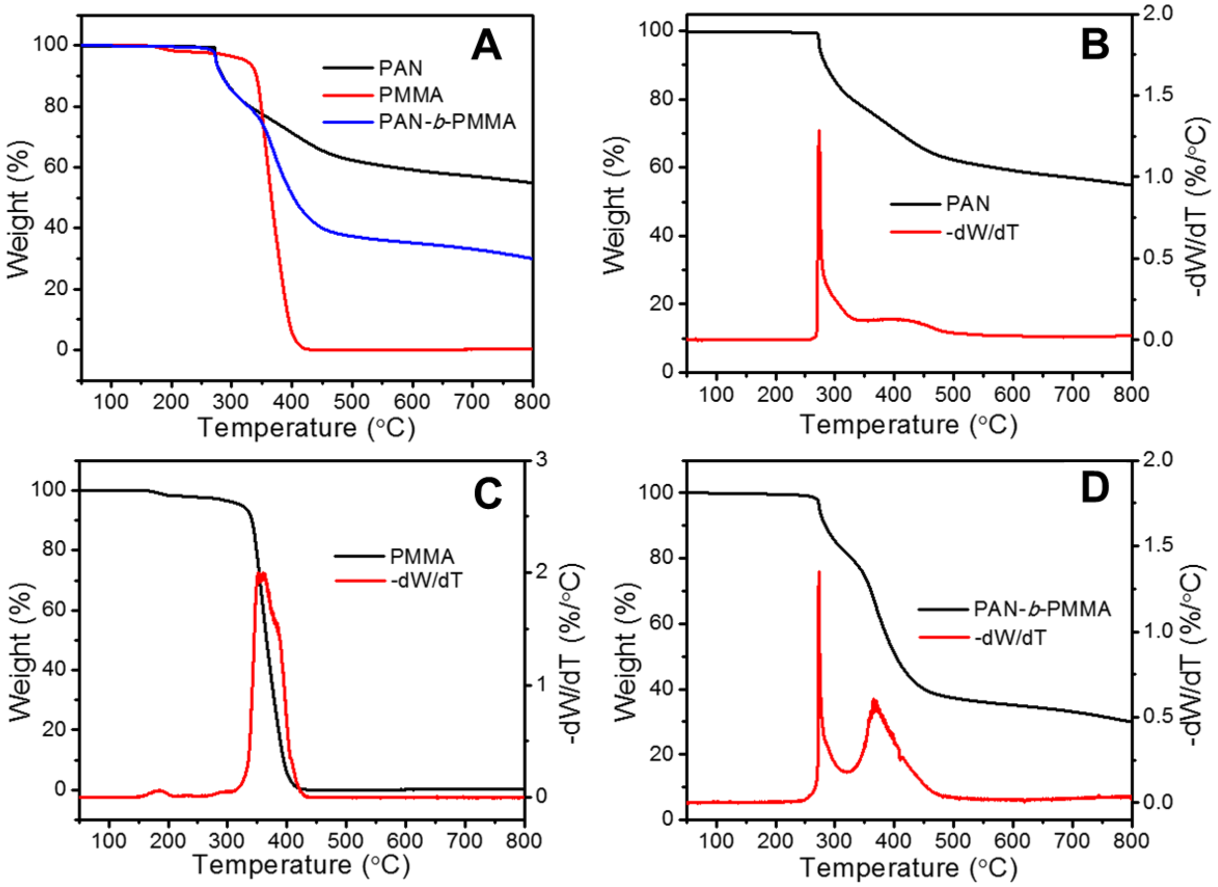

Fig. S2. Thermogravimetric analysis. (A) Thermogravimetric analysis (TGA) and the first derivative (labeled as –dW/dT) of the weight losses during the pyrolysis of (B) PAN, (C) PMMA and (D) PAN-*b*-PMMA. PAN-*b*-PMMA showed multiple weight loss stages including PAN stabilization (~250-320 °C), PMMA degradation (~320-430 °C), and carbonization of PAN at high temperatures.

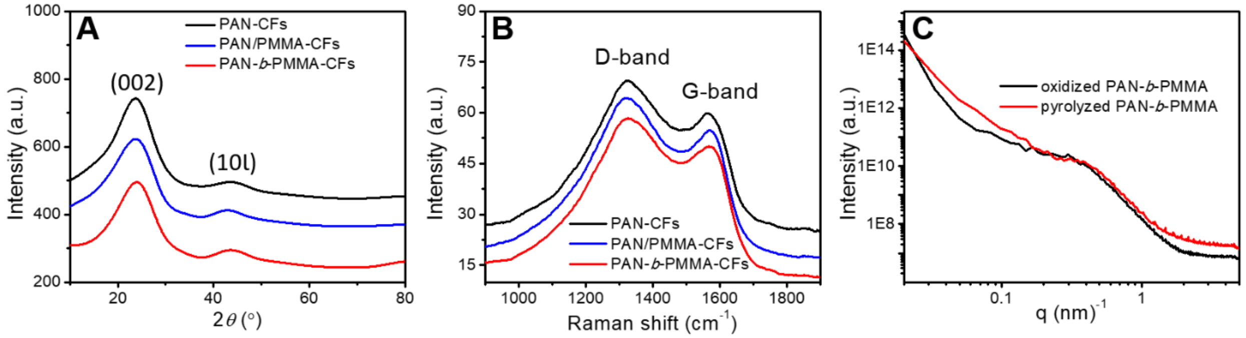

Fig. S3. Wide-angle XRD spectra, Raman spectra, and FFT spectra. (A) XRD profiles of PAN-CFs, PAN/PMMA-CFs and PAN-*b*-PMMA-CFs after pyrolysis at 800 °C. The diffraction peaks at ~24° and ~43° were assigned to the carbon crystallographic planes (002) and (10l), respectively. (10l) denotes the potentially overlapping peaks of (100) and (101) [*Carbon* **44**, 1986-1993 (2006)]. (B) Raman spectra of porous carbon fibers from PAN, PAN/PMMA and PAN-*b*-PMMA after pyrolysis at 800 °C. After pyrolysis, all the carbon fibers exhibited two characteristic carbon bands, *i.e.*, “G-band” at ~1560-1600 cm^{-1} and “D-band” at ~1310-1350 cm^{-1}. The calculated intensity ratio of “D-band” to “G-band” (I_D/I_G) is summarized in table S2. (C) Fast Fourier transform (FFT) spectra extracted from the SEM images of PAN-*b*-PMMA fibers after oxidation at 280 °C (black) and after pyrolysis at 800 °C (red).

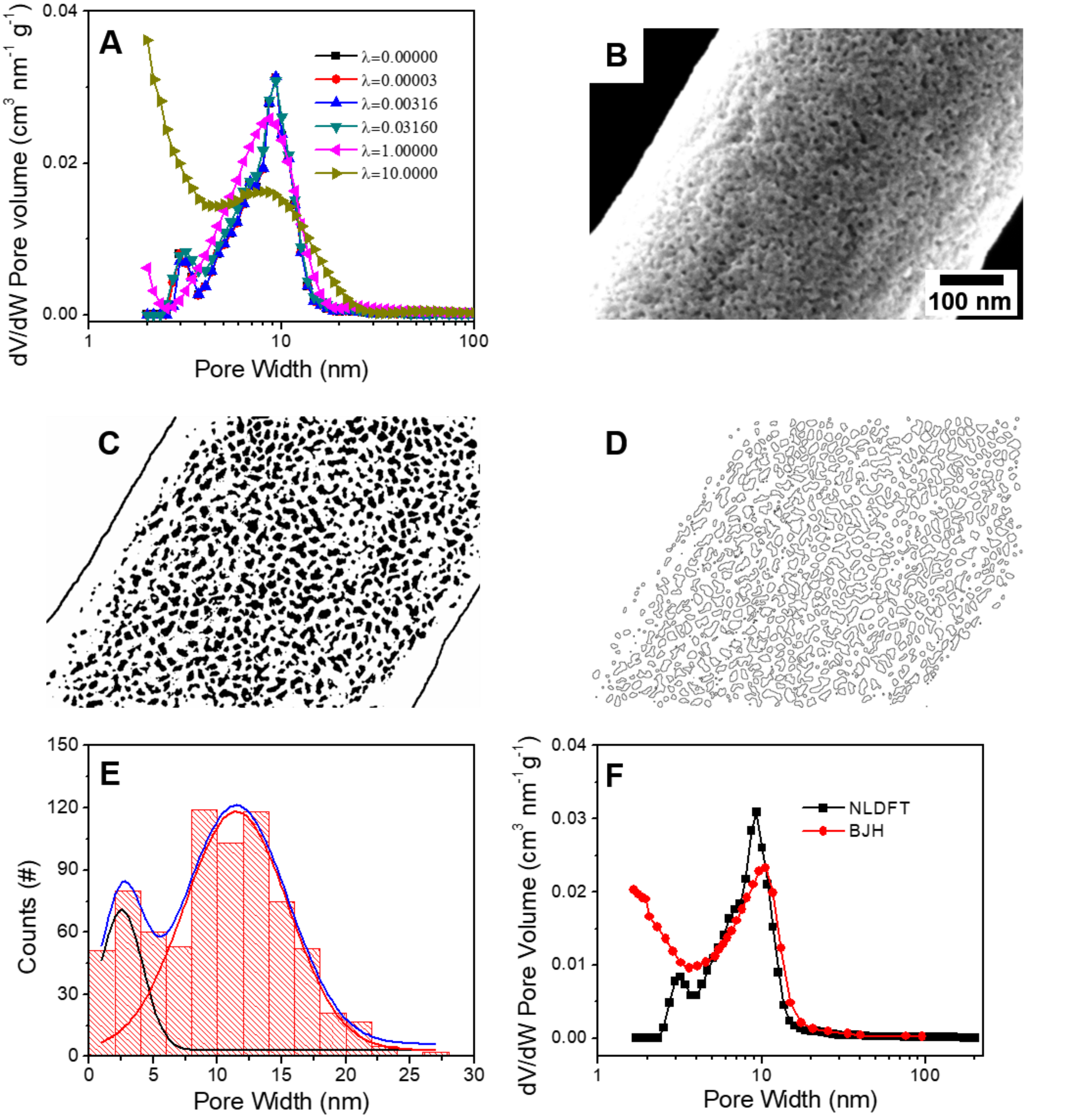

Fig. S4. Comparison of the pore size distributions from image analysis and NLDFT fitting. (A) PSDs from the NLDFT model when the regularization parameter (λ) was varied from 0 to 10. (B-E) Calculation of the pore sizes in PAN-*b*-PMMA-CF using image analysis. (B) An original SEM image, (C) a binary image of the SEM micrograph in black and white, and (D) identified boundaries of the pores. (E) PSD of PAN-*b*-PMMA-CF determined by ImageJ. The PSD curve (blue) was obtained through Gaussian fitting of two peaks (black and red). (F) Comparison of the PSDs of the PAN-*b*-PMMA-CFs using the NLDFT and BJH models.

As described in the report by Kupgan *et al.*, [*Langmuir* **33**, 11138-11145 (2017)], PSDs can be calculated using the following equation

$$N_{exp}(P/P_O) = \int_{D_{min}}^{D_{max}} N_{NLDFT}(P/P_O, D)\, PSD(D) dD + \lambda \int_{D_{min}}^{D_{max}} [PSD''(D)]^2 dD \qquad (18)$$

where N_{exp} is the experimental N_2 adsorption at 77 K; P/P_O is the relative pressure ratio; D is the pore diameter; N_{NLDFT} is the theoretical N_2 isotherm. The accuracy of the PSDs from the above NLDFT model depends on a regularization parameter, λ. Typically, higher λ values yield broader PSDs with less distinct peaks and thus lead to more artifacts. To evaluate the accuracy of NLDFT analysis and the effect of λ on the PSDs in our work, we have tuned λ from 0 to 10. As shown in fig. S4A, the PSDs do not change drastically when λ is in the range of 0-0.0316. However, the PSDs become broader when λ is increased to 1 and above. Thus, the PSDs using a λ value of 0.0316 should represent a reasonably good approximation to the actual PSDs of the porous carbon fibers.

To conduct the image analysis, a representative SEM image (fig. S4B) of a single PAN-*b*-PMMA-CF fiber is converted to a binary image in black-white mode (fig. S4C). The black areas in fig. S4C represent the pores. The boundaries of the pores are then identified and shown in fig. S4D. Assuming that the pore openings are circles, the pore sizes (*d*) can be calculated from the pore areas (*S*) according to the following equation

$$d = 2\sqrt{S/\pi} \qquad (19)$$

The Barrett-Joyner-Halenda (BJH) model is also commonly used for fitting mesoporous structures, and thus it is used to analyze the porous structures of our PAN-*b*-PMMA-CFs. As shown in fig. S4F, the PSD curve of PAN-*b*-PMMA-CFs obtained from the BJH model is similar to that from the NLDFT model in the pore size range >4 nm. The two fitted PSDs differ in the pore size range <4 nm. The shoulder peak at ~3-4 nm is absent in the PSD from the BJH model. Because the PSD from the NLDFT model matches with that from the image analysis, we have chosen NLDFT as the final model for pore size determination.

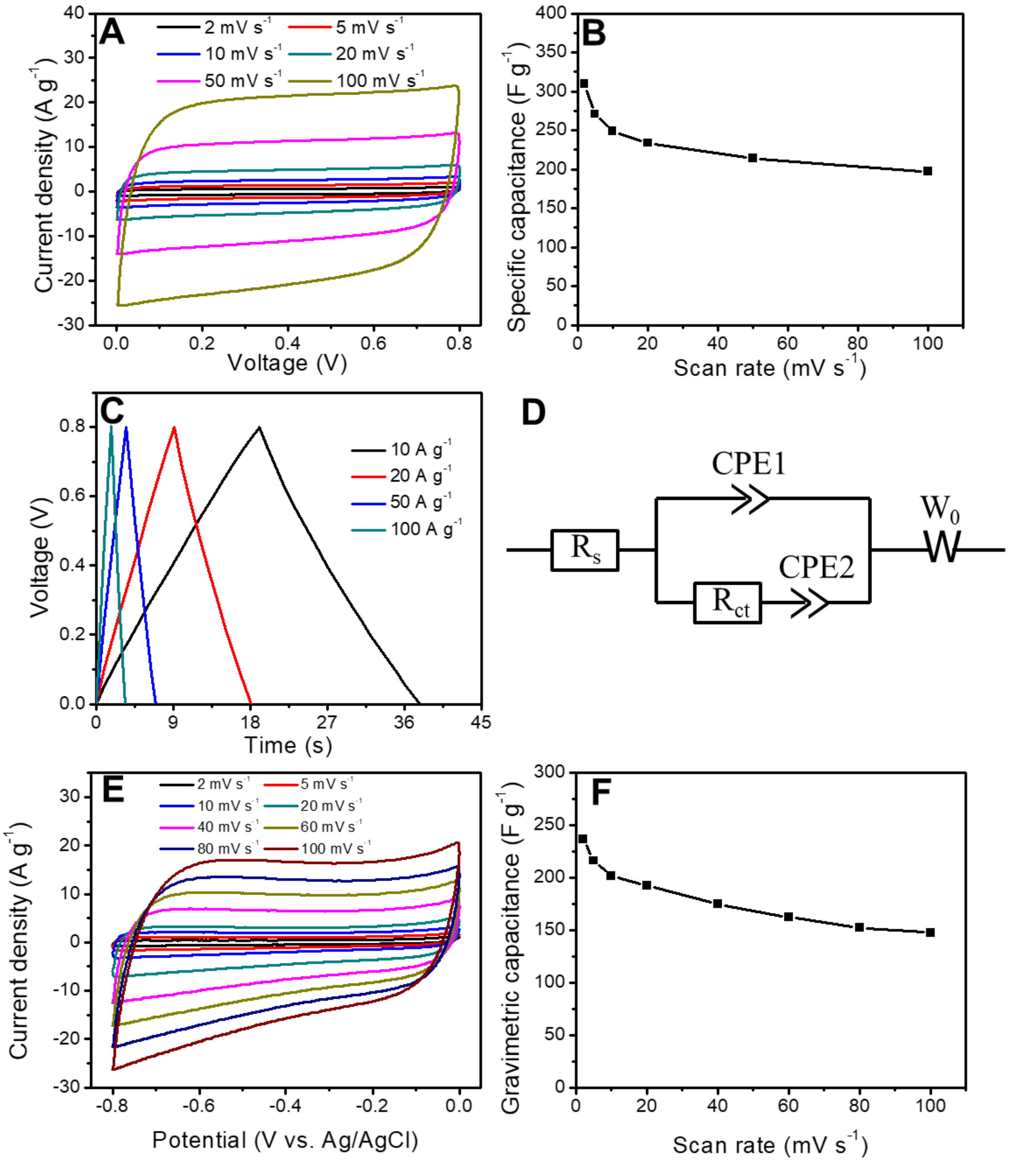

Fig. S5. Additional electrochemical performance of PAN-*b*-PMMA-CFs. (A to C) Electrochemical capacitive performance of a representative two-electrode cell made of PAN-*b*-PMMA-CFs. (A) Cyclic voltammograms (CV) at scan rates from 2 to 100 mV s^{-1}; (B) Specific gravimetric capacitance as a function of the scan rate; (C) Chronopotentiometry (CP) curves at current densities from 10 to 100 A g^{-1}. (D) An equivalent circuit model for fitting the Nyquist plots of PAN-*b*-PMMA-CFs. (E-F) Electrochemical capacitive performance of PAN-*b*-PMMA-

CFs evaluated by a three-electrode cell. (E) CV curves at scan rates from 10 to 100 mV s^{-1}. (F) The gravimetric capacitance as a function of scan rate.

For accuracy, constant phase elements (CPE) instead of ideal capacitors are used in the equivalent circuit model [*Electrochim. Acta* **115**, 587-598 (2014)]. The ions diffuse from the bulk electrolyte to both the carbon surface (double-layer capacitance, CPE1) and the heteroatoms (pseudocapacitance, CPE2). Thus, the Warburg impedance (W_0, the ion diffusion resistance) and the equivalent series resistance (R_s, the combination of the electrolyte resistance, the internal electrode resistance, and the interface resistance between the electrodes and the current collectors) are placed in series with the two capacitors, CPE1 and CPE2. Note that CPE1 is parallel to CPE2 because of their independent charge storage processes. For the pseudocapacitance CPE2, the redox electrochemical reaction is controlled by the kinetics of the charge transfer at the electrode-electrolyte interface, in other words, how fast the charges are transferred from the electrolyte to the electrode surface. Therefore, a charge transfer resistance (R_{ct}) is connected in series with CPE2 to describe the charge storage process associated with the heteroatoms.

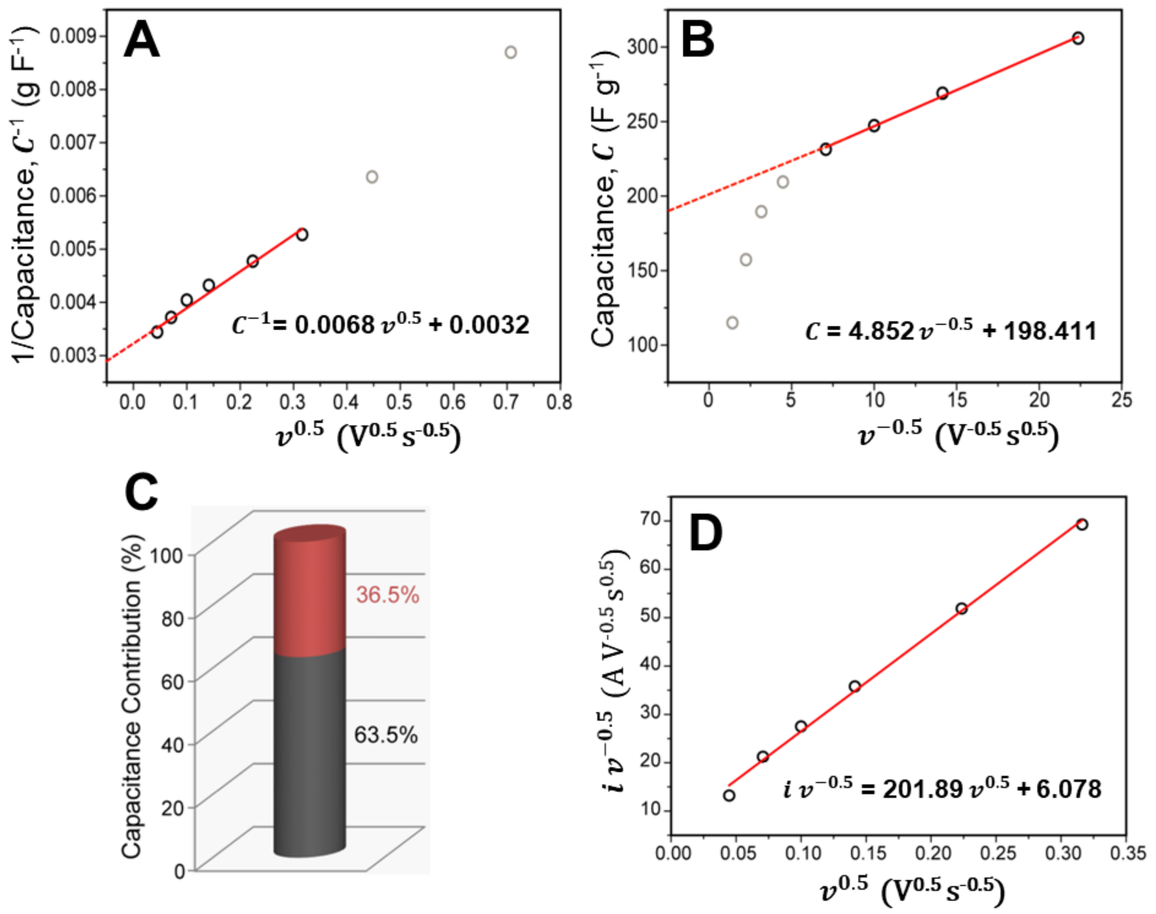

Fig. S6. Capacitance contribution analyses. (A to C) Trasatti's method analysis: (A) The reciprocal of gravimetric capacitance (C^{-1}) versus the square root of scan rate ($v^{0.5}$). (B) The gravimetric capacitance (C) versus the reciprocal of the square root of scan rate ($v^{-0.5}$). The red lines are the linear fittings to the data points at the low scan rates. The fitting equations are shown in the insets. Data points in grey are masked during the fitting. (C) Histogram showing the capacitance contributions from the capacitive processes (*i.e.*, electrical double-layer capacitance, black) and the diffusion-controlled processes (*i.e.*, pseudocapacitance, red). (D) Dunn's method analysis: $i\,v^{-0.5}$ vs. $v^{0.5}$ plot for PAN-*b*-PMMA-CFs using the anodic current at a potential of 0.5 V vs. Ag/AgCl.

Trasatti's Method

We analyzed the CV curves and the corresponding gravimetric capacitances (*C*) of PAN-*b*-PMMA-CFs at scan rates ranging from 2 to 100 mV s^{-1}. The reciprocal of gravimetric capacitances (C^{-1}) should scale linearly with the square root of scan rates ($v^{0.5}$), assuming ion diffusion follows a semi-infinite diffusion pattern (fig. S6A) [*J. Am. Chem. Soc.* **134**, 14846-14857 (2012)]. Specifically, the correlation can be described by the following equation

$$C^{-1} = 0.0068\, v^{0.5} + {C_T}^{-1} \tag{20}$$

where C_T is total capacitance. Data points at higher scan rates deviate from the relationship due to the intrinsic resistance of the electrode and the deviation from semi-infinite ion diffusion [*ACS Nano* **7**, 1200-1214 (2013)]. These deviated data points were masked during the linear fitting. C_T equals the sum of electrical double-layer capacitance and pseudocapacitance [*J. Power Sources* **227**, 300-308 (2013)].

Similarly, assuming a semi-infinite ion diffusion, the capacitance *C* follows a linear relationship with the reciprocal of the square root of scan rates ($v^{-0.5}$) (fig. S6B), as described by the following equation [*Electrochim. Acta* **35**, 263-267 (1990)]

$$C = 4.852\, v^{-0.5} + C_{EDL} \tag{21}$$

where C_{EDL} is the electrical double-layer capacitance. A linear fitting to the plot and extrapolation of the fitted line to the y-axis gives the maximum C_{EDL} [*J. Power Sources* **227**, 300-308 (2013)]. Subtraction of C_{EDL} from C_{T} yields the maximum pseudocapacitance. The histogram shows the percentages of C_{EDL} (63.5%) and pseudocapacitance (36.5%) (fig. S6C), respectively.

Dunn's Method

Dunn's method enables one to differentiate quantitatively the capacitance contributions from the surface capacitive effects (*i.e.*, EDL capacitive effects) and the diffusion-controlled processes (*i.e.*, pseudocapacitive reactions) [*J. Phys. Chem. C* **111**, 14925-14931 (2007)]. At a fixed potential, the current density (*i*) from the CVs can be expressed as a combination of two terms, *i.e.*

$$i = \mathrm{k}_1 v + \mathrm{k}_2 v^{0.5} \tag{22}$$

where the first term $k_1 v$ accounts for the current density contributed from the EDL capacitive effects while the second term $k_2 v^{0.5}$ is the current density associated with the pseudocapacitive reactions. Dividing $v^{0.5}$ on both sides of the equation yields

$$iv^{-0.5} = k_1 v^{0.5} + k_2 \quad (23)$$

Therefore, by reading i from the CVs at a series of scan rates and then plotting $i\, v^{-0.5}$ vs. $v^{0.5}$, one expects to obtain a linear fitting line with a slope of k_1 and a y-intercept of k_2. Fig. S6D displays an example of an $iv^{-0.5}$ vs. $v^{0.5}$ plot collected for PAN-*b*-PMMA-CFs using the anodic current at a potential of -0.1 V. Using the k_1 and k_2 values in Eq. (23) allows one to differentiate the capacitance contribution from C_{EDL} and pseudocapacitance at the specific potential V and a selected scan rate, v.

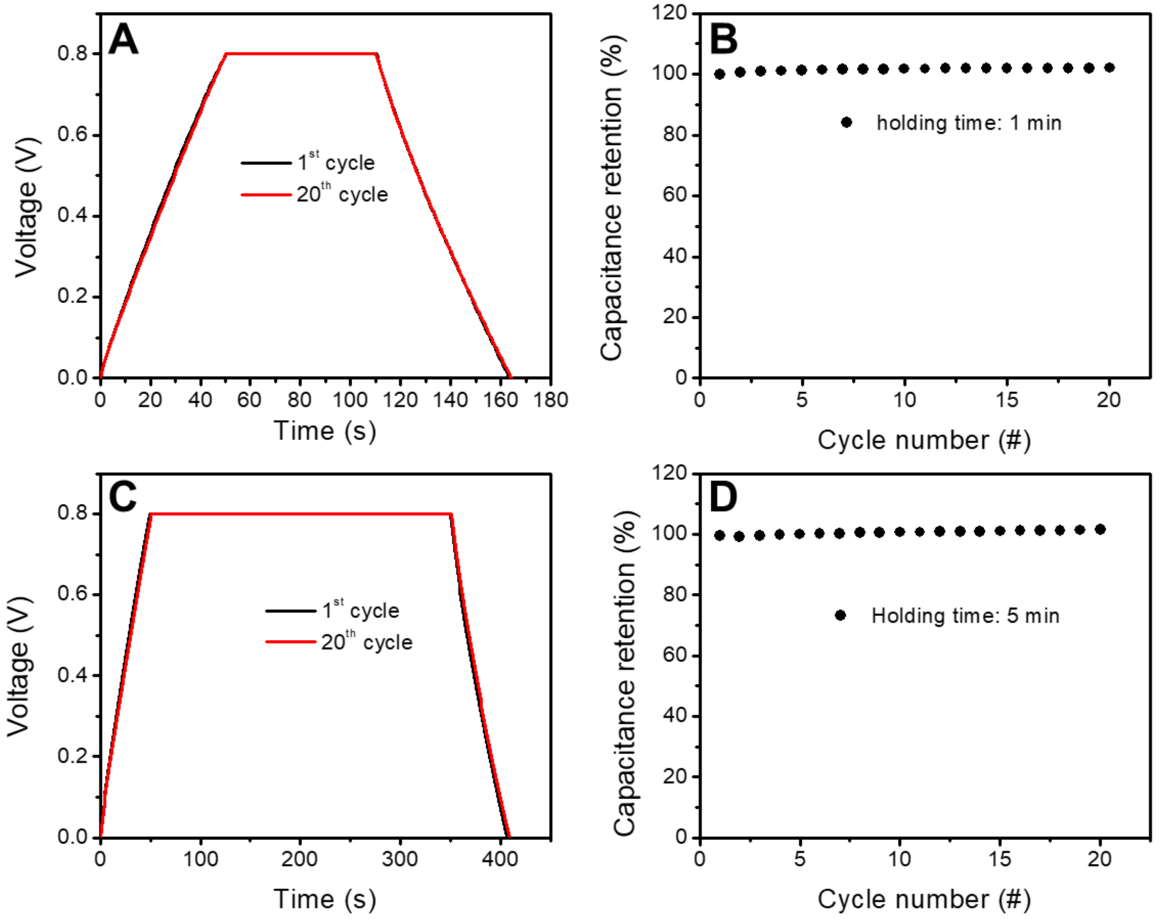

Fig. S7. Stability performance of PAN-*b*-PMMA-CFs. (A and C) Charge-hold-discharge voltage profiles of a PAN-*b*-PMMA symmetric supercapacitor in the 1st and 20th cycle with a holding time of (A) 1 min and (C) 5 min. (B and D) The capacitance retention of a PAN-*b*-PMMA symmetric supercapacitor with a holding time of (B) 1 min and (D) 5 min. The current density for the charge and discharge processes was set at 4 A g^{-1}.

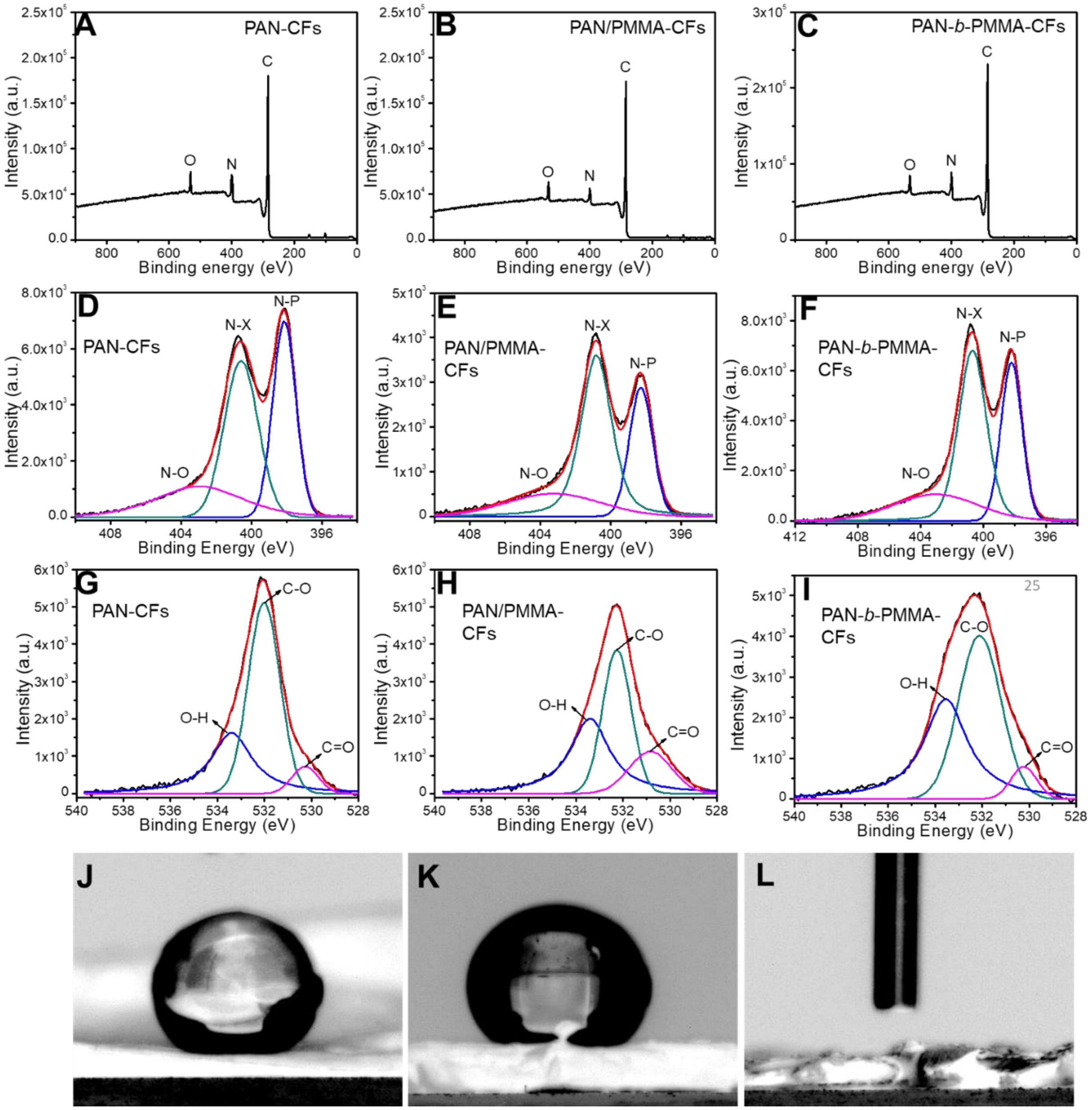

Fig. S8. XPS spectra and contact angles. XPS survey spectra of (A) PAN-CFs, (B) PAN/PMMA-CFs, and (C) PAN-*b*-PMMA-CFs. XPS N 1s peaks of (D) PAN-CFs, (E) PAN/PMMA-CFs, and (F) PAN-*b*-PMMA-CFs. XPS O 1s peaks of (G) PAN-CFs, (H) PAN/PMMA-CFs, and (I) PAN-*b*-PMMA-CFs. Contact angles of (J) PAN-CFs, (K) PAN/PMMA-CFs, and (L) PAN-*b*-PMMA-CFs after pyrolysis at 800 °C. The fluid used for testing was 6 M aqueous KOH.

Table S1. Summary of the electrochemical capacitive performance of PCF electrodes to generate the charts in Fig. 4. Note: 1) For fair comparison, the capacitances are all evaluated at 1 A g^{-1} in a symmetric two-electrode configuration. *Capacitances at other current densities are not considered.* 2) *For fair comparison of fibrous electrodes, the table only incudes capacitances of carbon fibers*. 3) The porous carbon fibers in this work are not activated, which can be done easily to further improve the performance. 4) No conductive additives or polymer binders are considered.

**Electrode materials**	**Precursors**	**Activation agents**	**Specific surface area (m^2 g^{-1})**	**Gravimetric capacitance (F g^{-1}, at 1 A g^{-1})**	**BET-area-normalized capacitance (μF cm^{-2})**	**References**
Porous flexible carbon nanofiber (CNF) paper	Phenolic resin/poly(vinyl alcohol) (PVA)	KOH	1317	235	18	*Chem. Eng. J.* 2014, 249, 216–225
CNFs with radially grown graphene sheets	PAN/graphene	NH_3	2185	133	6	*Nanoscale* 2013, 5, 4902–4909
Porous carbon nanofibers with carbon nanotube (CNT) fillers	PAN/CNT	H_2O_2	810	280	35	*J. Mater. Chem.* 2009, 19, 2810–2816
3D hierarchical porous carbon fibers	PAN	Hydrochloric acid	2177	~270	12	*J. Mater. Chem. A* 2015, 3, 14817-14825
Graphene integrating carbon fibers	PAN/graphene	KOH	830	129	16	*Carbon* 2018, 126, 500-506
Nomex-derived activated carbon fibers	Nomex aramid fibers	H_3PO_4/CO_2	2600	175 (5mV s^{-1})	7	*J. Power Sources* 2006, 153, 419-423
CNFs PAN	PAN	Water steam	1230	120	10	*Appl. Phys. Lett.* 2003, 83, 1216–1218
CNFs	PAN	CO_2	705	200	28	*Carbon* 2009, 47, 2984–2992
In-situ nitrogen-doped mesoporous CNFs	PAN	$Mg(OH)_2$	926	327	35	*J. Mater. Chem. A* 2017, 5, 23620–23627
Plasma oxidized electrospun CNFs	PAN	Plasma	274	160	58	*RSC Adv.* 2015, 5, 38868–38872
Activated carbon fiber webs	Poly(amic acid) (PAA)	Water steam	2100	175	8	*Electrochim. Acta* 2004, 50, 883–887
Porous CNFs	PAN/PMMA	CO_2	2419	140	6	*RSC Adv.* 2015, 5, 19865–19873
Porous CNFs	PAN	$ZnCl_2$	550	140	25	*Adv. Mater.* 2007, 19, 2341–2346
Activated porous CNFs using Sn	PAN/poly(vinylpyrrolidone) (PVP)	Sn/Acid	1082	175	16	*Carbon* 2013, 65, 87–96
Nitrogen-doped hierarchical porous carbon fibers	PAN/PMMA/ tetraethyl orthosilicate/ thermoplastic polyurethane	HF acid/SiO_2	1126	198	18	*J. Solid State Electrochem.* 2015, 19, 1591–1597
Nitrogen-doped hollow activated CNFs	PAN/PVP	NH_3	701	180	26	*J. Electroanal. Chem.* 2015, 739, 84–88

Hierarchical porous CNFs	PAN/PMMA/ tetraethyl orthosilicate (TEOS)	HF acid/SiO_2	699	170	24	*J. Solid State Electrochem.* 2013, 17, 2731–2739
Graphitic carbon nitride nanosheets @ N-enriched mesoporous CNFs	PAN/g-C_3N_4 nanosheets	Water steam	554	149	27	*Carbon* 2015, 94, 342–348
High surface-area CNFs	Synthesized polymer (PIM-1)	Water steam	1162	149	13	*J. Mater. Chem. A* 2014, 2, 418–424
Mesoporous CNFs with large cage-like pores	PVA/Sn-citric composite	Sn/HCl	800	103	13	*Carbon* 2014, 70, 295–307
N/P/K co-doped porous CNFs	Cane molasses	NA	580	172	30	*RSC Adv.* 2014, 4, 34739–34750
Porous CNFs	PAN/Nafion	NA	1499	210	14	*J. Power Sources* 2013, 235, 289–296
Heteroatom-enriched electrospun CNFs	Melamine formaldehyde resin/PVA	NA	320	160	50	*J. Colloid Interface Sci.* 2013, 395, 217–223
Electrospun CNFs/graphene	PAN/graphene	NA	480	183	38	*J. Power Sources* 2013, 243, 350–353
CNF/CNT composite	PAN/CNT/ spherical latex nanoparticles	NA	535	250	47	*Mater. Lett.* 2015, 161, 587–590
CNFs	Poly(amide imide)	NA	1360	100	7	*Mater. Sci. Eng. B* 2009, 164 , 106–111
Microporous CNFs	Phenolic resin/ TEOS/PVP	NA	2164	310	14	*Int. J. Hydrogen Energy* 2016, 41, 9383–9393
Nitrogen- and oxygen-enriched 3D hierarchical porous CNFs	PAN copolymer (acrylonitrile/ itaconic acid)	NA	2177	250	12	*J. Mater. Chem. A* 2015, 3, 14817–14825
Microporous CNFs	PAA/PVP	NA	804	180	22	*J. Power Sources* 2015, 278, 683–692
Nitrogen-enriched porous CNFs	PAN/allyl polyethylene glycol (PEG)	NA	753	270	36	*Electrochim. Acta* 2015, 158, 306–313
Porous CNTs(*55*)	Polyaniline nanotubes	NA	3253	285	9	*J. Mater. Chem. A* 2014, 2, 12545–12551

$ZnCl_2$-activated and filter paper-derived CNFs	Waste filter paper	$ZnCl_2$	2232	302	14	*RSC Adv*. 2015, 5, 72019–72027
Nitrogen-doped porous multi-nano-channel CNFs	PAN/ polystyrene	NA	840	325	39	*ACS Sustainable Chem. Eng*. 2016, 4, 2439–2448

Table S2. Summary of the physical and chemical characterization.

Characterizations			PAN-CFs	PAN/PMMA-CFs	PAN-*b*-PMMA-CFs
XRD	2θ (002)		23.8°	23.8°	24.0°
	2θ (10l)		43.5°	42.8°	43.5°
	β (002) (radian)		0.16	0.15	0.14
	β (10l) (radian)		0.21	0.11	0.11
	d_{002} (nm)		0.37	0.37	0.37
	lateral size, L_a (nm)		1.42	2.81	2.73
	crystallite size, L_c (nm)		0.86	0.92	0.98
SAXS	Index of the Porod's Law (x)		-3.37	-3.34	-3.43
	q (nm^{-1})		NA	NA	0.224
	Center-to-center spacing (nm)		NA	NA	28.5
Raman	D band center (nm)		1327	1325	1327
	G band center (nm)		1563	1572	1568
	I_D/I_G		1.16	1.16	1.16
BET	S_{BET} (m^2/g)		213	245	503
	S_{micro} (m^2/g)		190	185	348
	S_{meso} (m^2/g)		23	60*	155
	Mesopore size range (nm)		NA	2-200*	2-20
	Total pore volume (cm^3/g)		0.102	0.385	0.450
	Micropore volume (cm^3/g)		0.076	0.074	0.140
	Mesopore volume* (cm^3/g)		0.026	0.311*	0.310
XPS	C (%)		82.1±0.2	84.8±0.1	82.1±0.6
	N (%)		13.4±+0.4	9.8±0.7	12.8±0.1
	O (%)		4.6±0.2	5.4±0.6	5.0±0.6
	N-P	B.E. (eV)	398.1	398.3	398.2
		Content (%)	4.9±0.1	3.0±0.5	4.3±0.1
	N-X	B.E. (eV)	400.6	400.8	400.7
		Content (%)	5.5±0.2	4.7±0.2	5.8±0.1
	N-O	B.E. (eV)	403.0	403.2	403.0
		Content (%)	3.0±0.2	1.9±0.2	2.8±0.1
	C=O	B.E. (eV)	530.3	530.8	530.3
		Content (%)	0.5±0.1	0.6±0.2	0.4±0.1
	C-O	B.E. (eV)	532.0	532.3	532.1
		Content (%)	2.5±0.1	2.5±0.5	2.8±0.5
	O-H	B.E. (eV)	533.4	533.4	533.6
		Content (%)	1.6±0.1	2.2±0.3	1.8±0.1

**Electrical Properties**	Equivalent series resistance R_s (Ω)	1.37	1.27	1.00
	Charge transfer resistance R_{ct} (Ω)	3.43	2.95	1.49
	Diffusion resistivity σ (Ω $s^{-0.5}$)	2.01	1.78	0.87
	Bulk electrical resistivity ρ (Ω·cm)	18.44±1.57	14.12±0.69	6.83±0.27

Note:
*The mesopore surface area and volume were determined by subtracting the micropore surface area and volume from the total pore surface area and volume, respectively. The porous carbon fibers derived from PAN and PAN-*b*-PMMA had negligible macropores, while those from PAN/PMMA blends contained a substantial amount of macropores, as evident in both the SEM images and the pore-size distribution profiles. Therefore, the mesopore volume of 0.311 cm^3/g of PAN/PMMA-CFs included the contributions from both mesopores (55.4%) and macropores (44.6%). The percentages were estimated using NLDFT.
